# Coworking spaces vs. home: Does employees' experience of the negative aspects of working from home predict their intention to telework in a coworking space?

**DOI:** 10.3389/fpsyg.2022.1079691

**Published:** 2022-12-08

**Authors:** Colin Lescarret, Céline Lemercier, Valérie Le Floch

**Affiliations:** CLLE, Université de Toulouse, CNRS, Toulouse, France

**Keywords:** teleworking, teleworking location, coworking spaces, attitude, behavioral intention, job satisfaction

## Abstract

In this study, we investigated the determinants of employees' intention to telework in a coworking space, with the assumption that employees' experience with the negative aspects of teleworking from home would impact their intention to telework in a coworking space in the future. A sample of 268 French teleworkers answered an online questionnaire measuring their experience of several negative aspects of teleworking from home (e.g., perceived social isolation), and their opinion toward teleworking in a coworking space (perceived usefulness, perceived feasibility, attitude and behavioral intention). Results indicate that perceived social isolation and perceived lack of working comfort when working from home directly impacted how useful participants perceived teleworking in a coworking space to be, and indirectly their intention to telework in a coworking space in the future. Budget, management agreement and job compatibility were, however, identified as factors mitigating participants' intention to telework in a coworking space, even if perceived as potentially beneficial.

## Introduction

Advances in information and communication technologies and, more recently, the COVID-19 health crisis, have contributed to the democratization of teleworking among company employees (Mann and Holdsworth, 2003; Gajendran and Harrison, 2007; Vayre and Pignault, 2014; Vayre, 2019; Wang et al., 2021). Teleworking (also referred to as remote working, telecommuting) is broadly defined as “an alternative work arrangement in which employees perform tasks elsewhere that are normally done in a primary or central workplace, for at least some portion of their work schedule”, using ICTs to communicate with colleagues and others outside the organization (Gajendran and Harrison, 2007, p. 1525). Although home is the most common location for teleworking (Gajendran and Harrison, 2007; Vayre and Pignault, 2014; Wang et al., 2021), an increasingly popular location has emerged in the last decade, referred to as coworking spaces (Akhavan, 2021; Orel and Bennis, 2021).

Coworking spaces consist of alternative workplaces offering all the facilities necessary to work in good conditions (e.g., office equipment and internet connection), combined with areas dedicated to relaxation and exchange between users (Scaillerez and Tremblay, 2017; Akhavan, 2021; Orel and Bennis, 2021). The use of these spaces increased considerably in the 2010s, with an estimated number of 3.1 million users worldwide in 2022, and five million expected by 2024 (Statista, 2022a). While the primary users of coworking spaces are self-employed or freelance workers looking for an affordable place to work away from home, the proportion of employees teleworking is growing, reaching one third of users in 2019 (Deskmag, 2019). However, while the motivations of self-employed workers to use a coworking space have been the subject of several studies (Gerdenitsch et al., 2016; Orel, 2019; Robelski et al., 2019; Appel-Meulenbroek et al., 2021; Lashani and Zacher, 2021; Rådman et al., 2022), the reasons why employees may favor coworking spaces over home as a place to telework are largely unknown.

In this study, we investigated the determinants of employees' intention to telework in a coworking space, with the assumption that teleworkers' dissatisfaction with home-based teleworking (e.g., perceived social isolation) would impact their intention to telework in a coworking space. For this purpose, we conducted a questionnaire survey among a large sample of teleworkers, measuring their satisfaction with home-based teleworking and their opinion toward teleworking in a coworking space. Before presenting the study in greater detail, the relevant literature is reviewed.

Coworking spaces are considered to be part of a wider set of new urban spaces, referred to as third-places (Scaillerez and Tremblay, 2017; Akhavan, 2021; Orel and Bennis, 2021). The term “third-place” was initially introduced by Oldenburg (1989), to designate all places where workers could carry on their professional activity and gather outside their home (referred to as the “first-place”) and the company premises (“second-place”). Deriving from this definition, new forms of urban spaces identifying themselves as “third-places” have emerged from the 2000s onwards, some dedicated to creativity and innovation (e.g., FabLab), others to professional activity (e.g., business incubators).

Coworking spaces are a category of third-place dedicated to professional activity and aimed at nomadic workers (Scaillerez and Tremblay, 2017; Akhavan, 2021; Orel and Bennis, 2021). They offer the necessary facilities (e.g., desk, office chair, computer equipment, and internet connection) to carry out one's professional activity, upon payment of an access fee. These workspaces are most often combined with shared catering and relaxation areas (e.g., kitchen and sofas), in order to encourage interaction and the creation of social links between users (Akhavan, 2021; Orel and Bennis, 2021). Indeed, one of the main objectives of coworking space owners (referred to as “hosts”) is to encourage the creation of a community of users, through the organization of professional or informal events, which differentiates them from other shared workspaces such as flex offices (Orel and Bennis, 2021). In 2020, ~20,000 coworking spaces were in operation worldwide, and this number is expected to double by 2024 (Statista, 2022b).

While coworking spaces are open to all workers, regardless of their status and sector of activity, self-employed workers in the sector of ICT, marketing or consulting remain their primary users (Deskmag, 2019). According to several studies, the main reason for this population's interest in coworking spaces is to overcome a feeling of socio-professional isolation and to find social support (Spinuzzi, 2012; Gerdenitsch et al., 2016; Bianchi et al., 2018; Robelski et al., 2019; Spinuzzi et al., 2019; Lashani and Zacher, 2021; Rådman et al., 2022; Wright et al., 2022). Self-employed workers are at risk of experiencing a pronounced feeling of socio-professional isolation in the absence of colleagues to lean on if difficulties are encountered in the course of their work (Gerdenitsch et al., 2016). Joining a coworking space can help to break this feeling of isolation, by finding social support from other users of the space (occasional help with a task and collaboration) and regain the feeling of belonging, if not to a work group, at least to a community of users (Gerdenitsch et al., 2016; Bianchi et al., 2018; Lashani and Zacher, 2021; Wright et al., 2022). In this regard, Gerdenitsch et al. (2016) found that perceived social support from other coworking space users improved self-employed workers' job satisfaction and reduced their intention to quit their job.

In addition to finding social support, the use of a coworking space may help self-employed workers to better separate professional and private life, by relocating work outside the home and thus adding a physical separation between private life and work (Orel, 2019). The diversity of users' professional profiles also offers opportunities for knowledge sharing and professional networking which may help self-employed workers to expand their professional skills and activity (Spinuzzi, 2012; Spinuzzi et al., 2019; Rese et al., 2020). Finally, the mere provision of comfortable working facilities can enhance self-employed workers' productivity and health if their home workspace proves insufficient in terms of comfort or ergonomic qualities (Robelski et al., 2019, 2021; Lashani and Zacher, 2021; Rådman et al., 2022).

Although self-employed workers still constitute the majority of users of coworking spaces, the proportion of teleworking employees has become substantial. In a worldwide survey conducted by the magazine Deskmag in 2019, 37% of the 2,668 users of coworking spaces who answered the survey were teleworking employees (vs. 28% in the 2012 survey). To date, very little research has been conducted to understand why employees may decide to telework in a coworking space instead of (or alongside) home. There are, however, some negative consequences of teleworking that may contribute to explaining this “relocation” of work.

The advantages and disadvantages of teleworking have been the topic of a large number of studies during the last decades (Bailey and Kurland, 2002; Mann and Holdsworth, 2003; Gajendran and Harrison, 2007; Garrett and Danziger, 2007; Vayre and Pignault, 2014; Vayre, 2019), all the more so following the COVID-19 crisis and the switch of a significant proportion of employees to “forced” teleworking (Contreras et al., 2020; Bobillier-Chaumon et al., 2021; Wang et al., 2021). Despite this substantial body of work, there is currently no clear consensus on the impact of teleworking on employee performance and quality of life (Bailey and Kurland, 2002; Mann and Holdsworth, 2003; Garrett and Danziger, 2007; Vayre and Pignault, 2014; Vayre, 2019).

Several studies claim that teleworking has positive consequences on employees' productivity, in that teleworking contributes to reducing interruptions and distractions experienced in the office and thus improves employees' concentration and commitment at work (Bailey and Kurland, 2002; Mann and Holdsworth, 2003; Garrett and Danziger, 2007; Maruyama et al., 2009; Vayre and Pignault, 2014; Vayre, 2019). The flexibility in deciding how to organize one's working day (made possible by the elimination of time spent commuting) may also have a positive impact on work-life balance, by enabling employees to prioritize their activities and be more available for personal or family activities (Metzger and Cléach, 2004; Garrett and Danziger, 2007; Maruyama et al., 2009; Vayre and Pignault, 2014; Vayre, 2019). As a result, employees who telework on a regular basis may perceive an improvement in their job satisfaction and quality of life (Bailey and Kurland, 2002; Garrett and Danziger, 2007; Vayre and Pignault, 2014; Vayre, 2019).

Other studies, however, have highlighted that teleworking may have negative consequences, notably when done from home (Bailey and Kurland, 2002; Mann and Holdsworth, 2003; Metzger and Cléach, 2004; Maruyama et al., 2009; Vayre and Pignault, 2014; Vacherand-Revel et al., 2016; Vayre, 2019; Bobillier-Chaumon et al., 2021). As home becomes the second workplace, teleworkers may experience an overlap in space and time between their work life and their private life in the same way as the self-employed working from home (Vayre and Pignault, 2014; Vacherand-Revel et al., 2016; Orel, 2019; Vayre, 2019). This overlap can have a detrimental impact, first, on productivity by generating distractions and interruptions different from those experienced in the office, such as the need to care for young children or to carry out household tasks (Wang et al., 2021). It can also be detrimental for work-life balance and generate stress, if employees find themselves struggling to respond to both work and new family demands and free up time for themselves (Metzger and Cléach, 2004; Maruyama et al., 2009; Vayre and Pignault, 2014; Vacherand-Revel et al., 2016; Vayre, 2019). Despite the contribution of remote communication tools, the reduction in exchanges with colleagues imposed by distance can generate a feeling of solitude, erosion of the work group, and in some cases, social isolation, similar to that often experienced by self-employed workers (Cooper and Kurland, 2002; Gajendran and Harrison, 2007; Boboc et al., 2014; Vayre and Pignault, 2014; Vayre, 2019). Finally, employees may experience problems of working comfort (and over time, physical health) if their home workspace (office furniture, computer equipment) is inadequate (Robelski et al., 2019, 2021; Wang et al., 2021).

Overall, when the right working conditions are not met, the home may be a unsuitable place to telework (Müller et al., 2022). The health crisis linked to COVID-19, and the changeover of a significant part of the employees to “forced” telework has highlighted that some employees had a home unsuitable for work, due to lack of equipment or unfavorable family situations (Babic et al., 2021; Wang et al., 2021). Yet, workplace suitability is determinant for a successful transition to teleworking (Wang et al., 2021; Müller et al., 2022). Apart from the consequences in terms of wellbeing at work described above, Müller et al. (2022) recently demonstrated that workplace suitability is positively associated with work performance and collaboration, in the context of transition to teleworking. These results highlight that the workplace plays an important role in the success of teleworking deployment. When home proves to be an unsuitable place to telework, employees may need to look for an alternative workplace, such as coworking spaces.

In an exploratory study which remains, to our knowledge, the only one to have directly investigated teleworkers' opinions of coworking spaces (Lescarret et al., 2022), we conducted interviews with 20 employees teleworking on a regular basis (eight of whom were coworking space users) and questioned them on the perceived advantages and disadvantages of teleworking in coworking spaces. The results of these interviews showed that participants perceived coworking spaces as mainly useful for: (1) breaking the loneliness caused by teleworking at home and meeting new people, (2) improving productivity by eliminating sources of interruptions/distractions at home, (3) improving working comfort, and (4) separating private and professional life better. The cost of the access fees and the fear of an increase in commuting time were also identified as potential barriers to use. These results have yet to be replicated elsewhere, however, and the extent to which employees' experience of teleworking from home can predict their intention to telework in a coworking space remains to be investigated in more detail.

The present study aimed at better understanding the determinants of employees' intention to telework in a coworking space, and more specifically to what extent the experience of the negative aspects of teleworking from home (e.g., lack of social interaction at work) might contribute to this intention. To this end, we designed a survey measuring teleworkers' satisfaction regarding teleworking from home, and their opinion toward teleworking in a coworking space (perceived usefulness, perceived feasibility, attitude, and behavioral intention). This survey was administered to a large sample of French teleworkers who were not currently users of a coworking space, to investigate whether their experience of the negative aspects of teleworking from home influenced their intention to telework in a coworking space in the future.

Indeed, according to several theories of human behavior prediction, including the Theory of Planned Behavior (Ajzen, 1991, 2020), the intention to adopt a certain behavior is a function of one's attitude toward this behavior, i.e., how favorable or unfavorable one is toward adopting the behavior. This attitude is impacted, in turn, by beliefs regarding the likely consequences of this behavior (Ajzen, 1991, 2020) and notably, whether one would benefit from adopting the behavior, i.e., the behavior's perceived usefulness (Davis, 1989; Legris et al., 2003; King and He, 2006). Our main assumption was that the experience of the negative aspects of teleworking from home would impact how useful employees perceive teleworking in a coworking space to be, their attitude toward it, and ultimately their intention to telework in a coworking space in the future—the remaining question being which negative aspects and to what extent.

**Figure 1:**
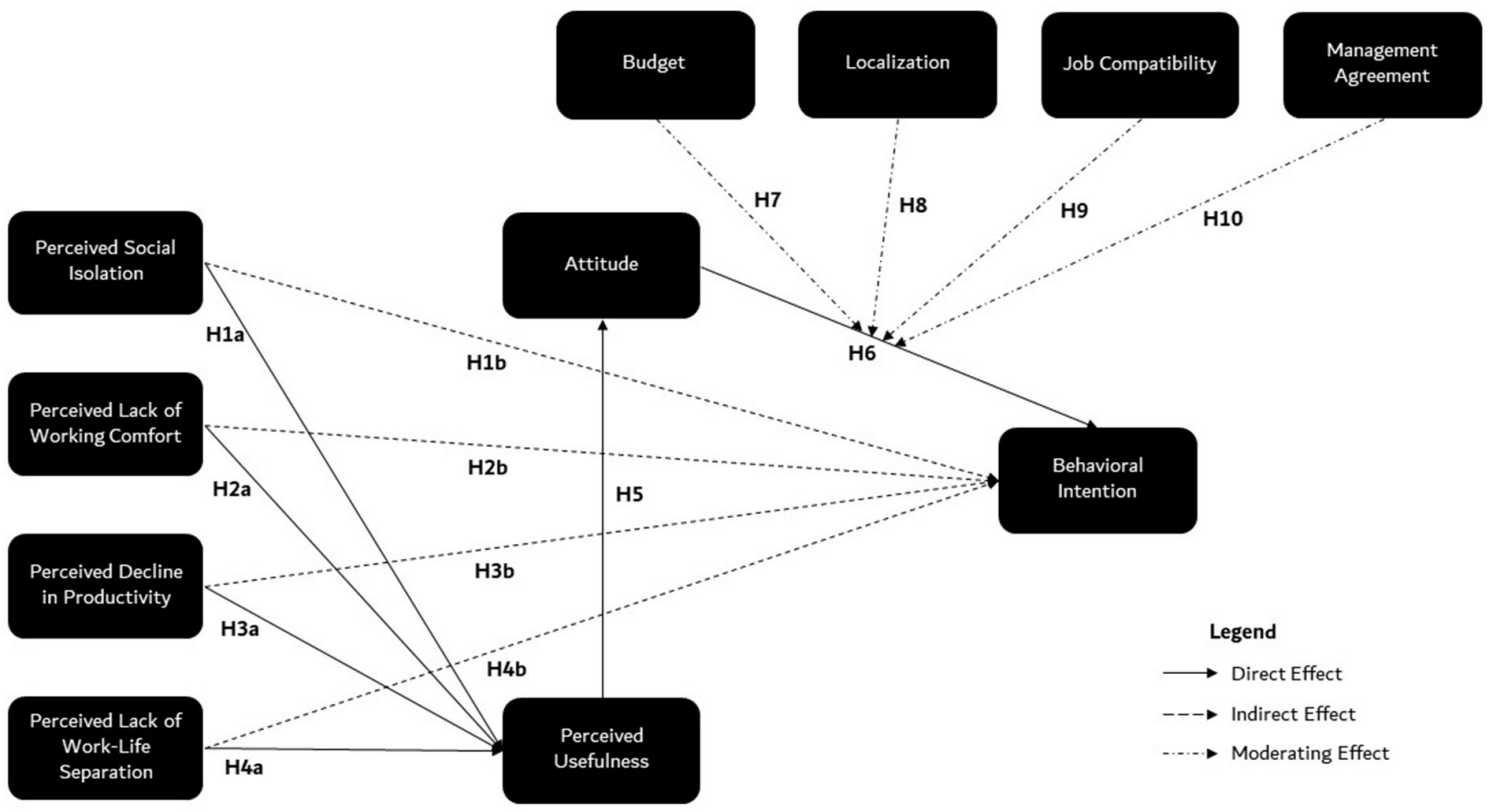
Theoretical model of the expected effects.

Based on the literature on teleworking and our pilot study (Lescarret et al., 2022), we identified four negative aspects of teleworking from home that might affect employees' intention to telework in a coworking space: (1) perceived social isolation (Cooper and Kurland, 2002; Gajendran and Harrison, 2007; Boboc et al., 2014; Vayre and Pignault, 2014; Vayre, 2019), (2) perceived decline in productivity (Wang et al., 2021), (3) perceived lack of working comfort (Robelski et al., 2019, 2021; Wang et al., 2021), and (4) perceived lack of work-life separation (Metzger and Cléach, 2004; Maruyama et al., 2009; Vayre and Pignault, 2014; Orel, 2019; Vayre, 2019). As represented in Figure 1, we expected that teleworkers' experience of these negative aspects of teleworking from home would have a direct effect on their perception of the usefulness of teleworking in a coworking space, and an indirect effect on their intention to telework in a coworking space in the future, mediated by its effect on perceived usefulness and attitude toward teleworking in a coworking space. Our hypotheses were formulated as follows:

**H1:** *Perceived social isolation* when working from home (PSI) has a direct effect on the perceived usefulness of teleworking in a coworking space (PU) (H1a), and an indirect effect on the intention to telework in a coworking space in the future (BI), mediated by its effect on PU and attitude toward teleworking in a coworking space (ATT) (H1b).
**H2:** *Perceived decline in productivity* when working from home (PDP) have a direct effect on PU (H2a), and an indirect effect on BI, mediated by its effect on PU and ATT (H2b).
**H3:** *Perceived lack of working comfort* when working from home (PLWC) have a direct effect on PU (H3a), and an indirect effect on BI, mediated by its effect on PU and ATT (H3b).
**H4:** *Perceived lack of work-life* separation when working from home (PLWLS) have a direct effect on PU (H4a), and an indirect effect on BI, mediated by its effect on PU and ATT.
**H5:** PU has a direct effect on ATT.
**H6:** ATT has a direct effect on BI.

Additionally, we were interested in the factors that might hinder employees' intention to telework in a coworking space, even if employees had a positive attitude toward it. Research has shown that a positive attitude toward a certain behavior does not mean that this behavior is feasible, for a variety of reasons (Ajzen, 1991, 2020). In accordance with the literature on coworking spaces and our pilot study (Lescarret et al., 2022), we decided to test the influence of four factors that might moderate employees' intention to telework in a coworking space: (1) the ability to pay the access fee to the coworking space, i.e., budget, (2) the availability of a coworking space near home, i.e., localization, (3) the feasibility of carrying out work tasks in a coworking space, i.e., job compatibility, and (4), whether the management would agree to the employee teleworking in a coworking space, i.e., management agreement. As represented in Figure 1, we expected these factors to moderate the effect of participants' attitude toward teleworking in a coworking space on their intention to telework in a coworking space in the future, in such a way that the positive effect of attitude on behavioral intention would be higher when perceived feasibility was high (e.g., having a sufficient budget to pay the access fees) but lower when perceived feasibility was low. The hypotheses were formulated as follows:

*Budget* (H7)*, Localization* (H8)*, Job Compatibility* (H9), *and Management Agreement* (H10) moderates the positive effect of ATT on BI.

## Methods

### Sample

Two hundred and sixty-eight French company employees participated in the study (Mage = 36 years old, SD = 8.65, Minage = 22, Maxage = 64). Out of this sample, 58.2% of participants (*N* = 156) considered themselves as a woman, 41% (*N* = 110) as a man, and 0.7% (*N* = 2) as non-binary or transgender. 12.7% of participants (*N* = 34) teleworked occasionally (less than once a week), 44.4% (*N* = 119) regularly (once or twice a week), and 42.9% (*N* = 115) 50% of their working hours or more. Most participants had a high position in the company organization (e.g., executives and engineers) and all had higher education qualifications, which corresponds to the demographics of French teleworkers before the COVID-19 crisis (DARES, 2019). All participants stated that they knew what a coworking space is, to some extent (34.7%, *N* = 93) or completely (65.3%, *N* = 175), but none were users of a coworking space at the time of the study.

Data collection took place from April to June 2022. Participants were recruited through professional networks (e.g., mailing list) and social networks (e.g., LinkedIn). Participation was strictly voluntary, anonymous and the study protocol was approved by the Ethical Committee of the University of Toulouse before the data collection. The study was designed in full compliance with the ethical standards of the Declaration of Helsinki.

Table 1 summarizes the socio-demographic characteristics of the sample.

**Table 1:** Sample description.

**Category**		**%**	***N***
Age		*M* = 36.28	*SD =* 8.65
Gender	Woman	58.2%	156
	Man	41%	110
	Non-binary/transgender	0.7%	2
Level of Education	Upper secondary-school	3.4%	9
	Bachelor/master	87.3%	234
	Ph.D.	9.3%	25
Marital status	Single	23.9%	64
	In a relationship	35.4%	95
	Married	40.7%	109
With children?	No	61.9%	166
	Yes	38.1%	102
Company size	<10 employees	9.7%	26
	(10:249) employees	25.4%	68
	(249: 4,999) employees	31.8%	88
	>5,000 employees	32.1%	86
Position in the company	Low (e.g., administrative assistant)	2.2%	6
	Intermediate (e.g., technician and sales consultant)	17.2%	46
	High (e.g., administrative executive and engineer)	80.6%	216
Teleworking intensity	Low (once or twice a month)	12.7%	34
	Moderate (once or twice a week)	44.4%	119
	High (50% of working hours or more)	42.9%	115
Teleworking location	From home, exclusively Mostly from home, occasionally from other places (e.g., library)	84.3% 14.2%	226 42
Do you know what a coworking space is?	Yes, to some extent Yes, completely	34.7% 65.3%	93 175

### Measures

#### Home-based teleworking satisfaction

In order to assess the extent to which participants perceived their experience of teleworking from home as satisfactory (or unsatisfactory), a 16-item questionnaire was developed based on the interviews we conducted with teleworkers in our pilot study (Lescarret et al., 2022). The items tackled four dimensions: (1) perceived social isolation (e.g., “When I work from home, I tend to feel lonely”), (2) perceived decline in productivity (e.g., “When I work from home, I find it hard to concentrate on my work”), (3) perceived lack of working comfort (e.g., “When I work from home, my working comfort is insufficient”), (4) perceived lack of work-life separation (e.g., “When I work from home, I find it difficult to ‘switch off' from work”). Participants had to indicate to what extent they agreed with each statement provided, on a 7-point Likert scale ranging from “Totally disagree” (scored 1) to “Totally agree” (scored 7). Negative items (“When I work from home, I tend to feel lonely”) contrasted with reverse scaled positive items (“When I work from home, I manage to stay in touch with my colleagues”) to prevent the emergence of an acquiescence bias (Schriesheim and Hill, 1981).

Participants' answers to the questionnaire were subjected to a Confirmatory Factor Analysis and a Reliability Analysis (CFA), using the R packages psych (Revelle, 2019) and lavaan (Rosseel et al., 2018). After the removal of two items with unsatisfactory factor loadings, the CFA indicated an acceptable fit of the four-factor structure. While the exact fit test proved significant, χ^2^_(71)_ = 244.652, *p* < 0.001, which may be explained by the sample size (Gatignon, 2010), comparative fit indexes were indicative of an acceptable model fit, with CFI = 0.931, TLI = 0.91 (Hu and Bentler, 1999). Reliability indexes also proved satisfactory for each dimension (all α > 0.80). The scores obtained on each item selected on the basis of this analysis were summed and averaged for each dimension to obtain a score between 1 and 7. Table 2 provides the full list of items along with the factor loadings on each dimension.

**Table 2:** Dimensions and corresponding items of the teleworking satisfaction questionnaire.

**Factor**	**When I work from home…**	**Estimate**	**SE**	***Z***	***p***
Perceived social isolation (α = 0.82)	…I tend to feel lonely	1.360	0.1109	12.3	<0.001
	…I still feel part of a working group*	1.316	0.0758	17.4	<0.001
	…I manage to stay in touch with my colleagues*	1.278	0.0722	17.7	<0.001
Perceived decline in productivity (α = 0.91)	…I find it hard to motivate myself to work	1.727	0.0916	18.8	<0.001
	… I find it difficult to concentrate on my work	1.671	0.0903	18.5	<0.001
	… I feel I am more productive than usual*	1.349	0.0844	16.0	<0.001
Perceived lack of working comfort (α = 0.92)	…my working comfort seems insufficient	1.485	0.0930	16.0	<0.001
	… I find it difficult to find a suitable space to work	1.581	0.0909	17.4	<0.001
	… I have all the necessary equipment to work efficiently*	1.461	0.0928	15.7	<0.001
	… I have a dedicated workspace that I find comfortable*	1.654	0.0898	18.4	<0.001
Perceived lack of work-life separation (α = 0.85)	…I find it hard to 'switch off' from work	1.320	0.1015	13.0	<0.001
	… I can easily find time for myself (or my family)*	0.999	0.0814	12.3	<0.001
	… I am satisfied with the balance between my professional and private life*	1.418	0.0900	15.8	<0.001
	… I feel that my work is intruding on my private life	1.220	0.0883	13.8	<0.001

Items marked with a (*) correspond to reverse-scaled items.

#### Opinion toward teleworking in a coworking space

##### Perceived usefulness

After completing the teleworking satisfaction questionnaire, participants were provided with the following description of what a coworking space is: “*Coworking spaces are alternative workplaces dedicated to mobile workers (self-employed or teleworking employees). They offer the necessary facilities for professional practice (private or open-plan offices, meeting rooms, computer equipment), combined with spaces that are more conducive to relaxation and exchanges between users (sofas, restaurant space, etc.)*.

*It is possible to rent an office on a one-off basis (one day, for example) or on a more regular basis in the form of a monthly subscription. For example, the average rates for Toulouse are €15 (incl. tax) per day, €120 (incl. tax) for 10 days and €200/month (excl. tax) for unlimited use*.

*In addition to renting offices, the owners of these spaces regularly organize events aimed at encouraging exchanges between users, in the form of workshops to share practices, professional networking, or more informal events (yoga sessions, games, etc.)*.”

This description was provided in case participants were unsure what a coworking space was, and was written to be as neutral as possible, in order not to induce a positive (or negative) attitude toward coworking spaces based on the information provided.^1^

After reading the description, participants were asked to indicate to what extent they perceived that teleworking in a coworking space would be useful to them (“In my case, attending a coworking space to telework would be…”), on a 9-point bipolar scale ranging from −4 (“useless”) to +4 (“useful”).

##### Perceived feasibility

Participants were then asked to indicate, on a 7-point Likert scale ranging from “Totally disagree” (1) to “Totally agree” (7), to what extent they agreed with the following statements: “I have the budget to pay a subscription to a coworking space” (Budget), “I think I would easily find a coworking space close to my home” (Localization), “My job is feasible in a coworking space” (Job Compatibility), “I think my managers will agree to my working in a coworking space” (Management Agreement). The four items were found to be only weakly correlated with each other (see Appendix B) and were therefore considered separately in the analyses.

##### Attitude toward teleworking in a coworking space

Participants were then asked to answer a four-item questionnaire aimed at assessing to what extent they were in favor of (or against) teleworking in a coworking space. Participants had to indicate to what extent they agreed with statements such as (“I want to telework in a coworking space”), on a 7-point Likert scale ranging from “Totally disagree” (1) to “Totally agree” (7). The reliability of the scale proved to be very high (α = 0.95).

##### Behavioral intention

Finally, participants had to answer a four-item questionnaire designed to evaluate to what extent they intended to telework in a coworking space in the future. Participants had to indicate how far they agreed with statements such as (“I will enquire about coworking opportunities near me”), on a 7-point Likert scale ranging from “Totally disagree” (1) to “Totally agree” (7). The reliability of the scale proved to be excellent (α = 0.92).

Table 3 summarizes the set of scales used to assess participants' opinion of teleworking in coworking spaces.

**Table 3:** Items used to measure: (1) Perceived usefulness, (2) Perceived feasibility, (3) Attitude, and (4) Behavioral intention.

**Dimension**	**Items**
Perceived usefulness	“*In my case, attending a coworking space to telework would be…”* Useless (−4)—Useful (+4)
Perceived feasibility	“*I have the budget to pay a subscription to a coworking space*” (Budget)
	“*I think I would easily find a coworking space close to my home*” (Localization)
	“*My job is feasible in a coworking* space” (Job compatibility)
	“*I think my managers will agree to my working in a coworking space*” (Management agreement)
Attitude (α = 0.95)	“*If I had the opportunity, I would go to a coworking space to telework*”
	“*I want to telework in a coworking space*.”
	“*I am reluctant to telework in a coworking space*.”
	“*I am not interested in teleworking in a coworking space*.”
Behavioral Intention (α = 0.92)	“*I will enquire about coworking opportunities near me*”
	“*I intend to try teleworking in a coworking space for a day, to see if I like the experience”*
	“*I have no intention of attending a coworking space in the future”*
	“*I am seriously considering attending a coworking space to telework (occasionally or regularly).”*

### Statistical analyses

All analyses were run using Jamovi software version 2.2 (The Jamovi Project, 2021), with the R packages psych: Procedures for Psychological, Psychometric and Personality Research (Revelle, 2019), lavaan: Latent Variable Analysis (Rosseel et al., 2018), car: Companion to Applied Regression (Fox and Weisberg, 2020), emmeans: Estimated Marginal Means (Lenth, 2020), and jAMM: jamovi Advanced Mediation Models (Gallucci, 2020).

Age, gender, level of education, marital status, number of children, position in the company, company size, teleworking intensity, and teleworking location were considered as control variables in the analyses.

## Results

Appendix A provides the means, standard deviations, skewness and kurtosis coefficients observed on each measure. Appendix B indicates the correlations observed between the measures.

### Preliminary analyses

Age, gender, level of education, marital status, number of children, position in the company, company size and teleworking location proved not be significantly associated with the measures, and were thus not included in further analyses. Conversely, teleworking intensity was found to be associated with perceived social isolation, *F*_(2, 95.2)_ = 4.782, *p* = 0.010, perceived usefulness, *F*_(2, 98.8)_ = 4.448, *p* = 0.014, attitude, *F*_(2, 101.2)_ = 5.593, *p* = 0.005, and behavioral intention, *F*_(2, 102.2)_ = 13.086, *p* < 0.001. Table 4 indicates the results of the *post-hoc* tests (Games-Howell). Overall, employees who teleworked at least 50% of their working hours reported feeling more socially isolated when working from home, had a more positive attitude toward teleworking in coworking spaces, perceived it as more useful, and were more inclined to telework in a coworking space in the future than the other employees. As a result, teleworking intensity was considered as a potential moderator in the following analyses.

**Table 4:** Results of the *post-hoc* tests (Games-Howell) pertaining to the effect of teleworking intensity on perceived lack of social interaction, perceived usefulness, attitude, and behavioral intention.

	**Teleworking intensity level**		**Low**	**Moderate**	**High**
Perceived social isolation	Low (*M_*PSI*_* = 2.94, *SD_*PSI*_* = 1.27)	*M*_difference_	—	−0.196	−0.697
		*t*-value	—	−0.800	−2.63
		df	—	52.1	67.5
		*p*-value	—	0.705	0.028
	Moderate (*M_*PSI*_* = 3.13, *SD_*PSI*_* = 1.23)	*M*_difference_	—	—	−0.500
		*t*-value	—	—	−2.66
		df	—	—	213.5
		*p*-value	—	—	0.023
	High (*M_*PSI*_* = 3.63, *SD_*PSI*_* = 1.61)	*M*_difference_	—	—	—
		*t*-value	—	—	—
		df	—	—	—
		*p*-value	—	—	—
Perceived usefulness	Low (*M_*UP*_* = −1.47, *SD_*UP*_* = 2.44)	*M*_difference_	—	−0.252	−1.236
		*t*-value	—	−0.518	−2.44
		df	—	58.4	66.5
		*p*-value	—	0.863	0.045
	Moderate (*M_*UP*_* = −1.22, *SD_*UP*_* = 2.71)	*M*_difference_	—	—	−0.984
		*t*-value	—	—	−2.60
		df	—	—	226.8
		*p*-value	—	—	0.027
	High (*M_*UP*_* = −0.23, *SD_*UP*_* = 3.05)	*M*_difference_	—	—	—
		*t*-value	—	—	—
		df	—	—	—
		*p*-value	—	—	—
Attitude	Low (*M_*ATT*_* = 3.66, *SD_*ATT*_* = 1.46)	*M*_difference_	—	0.0231	−0.712
		*t*-value	—	0.0773	−2.36
		df	—	64.3	65.9
		*p*-value	—	0.997	0.054
	Moderate (*M_*ATT*_* = 3.64, *SD_*ATT*_* = 1.79)	*M*_difference_	—	—	−0.735
		*t*-value	—	—	−3.13
		df	—	—	231.6
		*p*-value	—	—	0.006
	High (*M_*ATT*_* = 4.37, *SD_*ATT*_* = 1.81)	*M*_difference_	—	—	—
		*t*-value	—	—	—
		df	—	—	—
		*p*-value	—	—	—
Behavioral intention	Low (*M_*BI*_* = 2.08, *SD_*BI*_* = 1.19)	*M*_difference_	—	−0.422	−1.262
		*t*-value	—	−1.76	−4.78
		df	—	60.2	81.2
		*p*-value	—	0.192	<0.001
	Moderate (*M_*BI*_* = 2.51, *SD_*BI*_* = 1.37)	*M*_difference_	—	—	−0.839
		*t*-value	—	—	−4.02
		df	—	—	213.5
		*p*-value	—	—	<0.001
	High (*M_*BI*_* = 3.36, *SD_*BI*_* = 1.79)	*M*_difference_	—	—	—
		*t*-value	—	—	—
		df	—	—	—
		*p*-value	—	—	—

### Hypotheses testing

**Figure 2:**
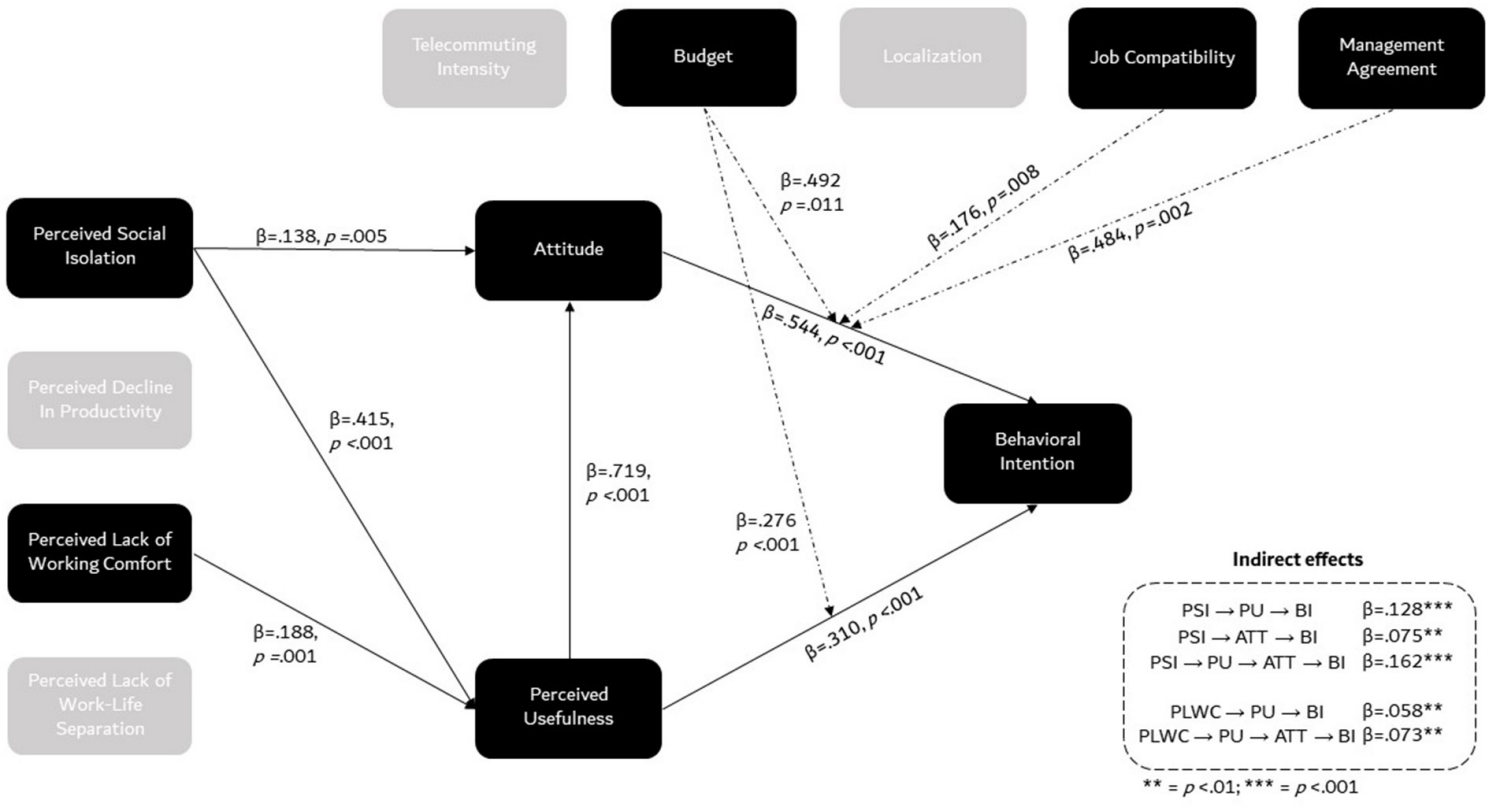
Overview of the results of the moderated mediation analysis.

We conducted a moderated mediation analysis, based on multiple linear regression modeling, to test our assumptions (Hayes, 2022). The R packages jAMM (Gallucci, 2020) and lavaan (Rosseel, 2019) were used to run the analysis. Behavioral Intention was included as the dependent variable; Perceived Social Isolation (PSI), Perceived Decline in Productivity (PDP), Perceived Lack of Working Comfort (PLWC), and Perceived Lack of Work-Life Separation (PLWLS) as covariate predictors; Perceived Usefulness and Attitude as mediators; Budget, Localization, Job Compatibility, and Company as moderators. Because of its significant association with several measures (including Behavioral Intention), Teleworking Intensity was also included as a potential moderator of the tested effects. Multicollinearity proved moderate enough to identify the effect of individual predictors, as VIF coefficients ranged between 1.067 (Budget) and 3.408 (Attitude) (Sheather, 2009). Figure 2 provides an overview of the results of the moderated mediation analysis.

#### Model components

In line with H1a, Perceived Social Isolation had a strong positive effect on Perceived Usefulness, β = 0.414, *z* = 6.342, *p* < 0.001, and (to a smaller extent) on Attitude toward teleworking in coworking spaces, β = 0.138, *z* = 2.785, *p* = 0.005. Perceived Lack of Working Comfort also had a significant positive effect on Perceived Usefulness, β = 0.188, *z* = 3.19, *p* = 0.001, in accordance with H3a, but its effect on Attitude was not significant, β = 0.026, *z* = 0.297, *p* = 0.766. Perceived Decline in Productivity had no significant effect on Perceived Usefulness, β = 0.094, *z* = 1.465, *p* = 0.143, nor on Attitude, β = 0.037, *z* = 0.82, *p* = 0.412, thus invalidating H2a. In discordance with H4a, Perceived Lack of Work-Life Separation also had no significant impact on Perceived Usefulness, β <0.001, *z* = 0.141, *p* = 0.989, or on Attitude, β = 0.035, *z* = 0.952, *p* = 0.341. No interaction effect between the covariates included as predictors were found on Perceived Usefulness, or on Attitude (all *p* > 0.10).

In line with the suspected mediated effects, Perceived Usefulness strongly and positively impacted Attitude, β = 0.791, *z* = 16.627, *p* < 0.001, and Behavioral Intention, β = 0.310, *z* = 4.518, *p* < 0.001. Attitude also proved to be a strong predictor of Behavioral Intention, β = 0.544, *z* = 8.013, *p* < 0.001. H5 and H6 were thus confirmed.

#### Direct and mediated effects on behavioral intention

None of the covariates included as predictors had a significant direct effect on Behavioral Intention. The direct effect of Perceived Lack of Work-Life Separation on Behavioral Intention was tendential, but small, and in the opposite direction to that expected, β = −0.080, *z* = −1.941, *p* = 0.052.

Although no significant direct effect of the covariates was found on Behavioral Intention, several indirect effects were noted. Perceived Social Isolation had a positive indirect effect on Behavioral Intention mediated by Perceived Usefulness, β = 0.128, *z* = 3.679, *p* < 0.001, and a positive indirect effect mediated by Attitude, β = 0.075, *z* = 2.631, *p* = 0.009. The full mediated path (PSI = > PU = > Attitude = > BI) also proved significant, β = 0.162, *z* = 4.672, *p* < 0.001. As a result, the total effect of Perceived Social Isolation on Behavioral Intention was found to be significant and large, β = 0.373, *z* = 5.08, *p* < 0.001. These results provide support for H1b.

In line with H3b, Perceived Lack of Working Comfort also had a positive (albeit small) indirect effect on Behavioral Intention, mediated by Perceived Usefulness, β = 0.058, *z* = 2.61, *p* = 0.009. Although the effect of Perceived Lack of Working Comfort on Behavioral Intention mediated by Attitude was not significant, β = 0.006, *z* = 0.297, *p* = 0.766, the full mediated path (PLWC = > PU = > Attitude = > BI) reached significance, β = 0.073, *z* = 2.924, *p* = 0.003. The total effect of Perceived Lack of Working Comfort on Behavioral Intention was moderate and positive, but only tendential, β = 0.122, *z* = 1.858, *p* = 0.063.

In discordance with H2b and H4b, however, no indirect effect of Perceived Decline in Productivity nor of Perceived Lack of Work-Life Separation was observed on Behavioral Intention, regardless of the mediators considered (all *p* > 0.10). Moreover, no indirect effect was found to be qualified with an interaction between the covariates. Table 5 recapitulates the results of the mediation analysis.

**Table 5:** Results of the mediation analysis.

**Type**	**Effect**	**Estimate**	**SE**	**95% CI**		**β**	***Z***	***p***
				**Lower**	**Upper**			
Indirect	PSI ⇒ UP ⇒ BI	0.1444	0.0392	0.0675	0.2213	0.1284	3.6796	< 0.001
	PLWC ⇒ UP ⇒ BI	0.0579	0.0222	0.0144	0.1013	0.0581	2.6104	0.009
	PDP ⇒ UP ⇒ BI	0.0283	0.0203	−0.0115	0.0681	0.0291	1.3942	0.163
	PLWLS⇒ UP ⇒ BI	2.75e-4	0.0195	−0.0379	0.0385	2.29e-4	0.0141	0.989
	PSI ⇒ ATT ⇒ BI	0.0846	0.0322	0.0216	0.1477	0.0753	2.6310	0.009
	PLWC ⇒ ATT ⇒ BI	0.0068	0.0230	−0.0389	0.0518	0.0068	0.2972	0.766
	PDP ⇒ ATT ⇒ BI	0.0198	0.0243	−0.0278	0.0674	0.0204	0.8157	0.415
	PLWLS⇒ ATT ⇒ BI	−0.0231	0.0244	−0.0709	0.0248	−0.0192	−0.9454	0.344
	PSI ⇒ UP ⇒ ATT ⇒ BI	0.1825	0.0383	0.1074	0.2576	0.1623	4.7646	<0.001
	PLWC ⇒ UP ⇒ ATT ⇒ BI	0.0732	0.0250	0.0241	0.1222	0.0735	2.9243	0.003
	PDP ⇒ UP ⇒ ATT ⇒ BI	0.0358	0.0249	−0.0130	0.0847	0.0368	1.4364	0.151
	PLWLS⇒ UP ⇒ ATT ⇒ BI	3.47e-4	0.0246	−0.0479	0.0486	2.90e-4	0.0141	0.989
Component	PSI ⇒ UP	0.8300	0.1309	0.5735	1.0865	0.4147	6.3423	<0.001
	PLWC ⇒ UP	0.3327	0.1040	0.1288	0.5366	0.1878	3.1984	0.001
	PDP ⇒ UP	0.1628	0.1111	−0.0549	0.3805	0.0941	1.4657	0.143
	PLWLS ⇒ UP	0.0016	0.1120	−0.2179	0.2210	7.40e-4	0.0141	0.989
	PSI ⇒ ATT	0.1725	0.0619	0.0511	0.2939	0.1383	2.7854	0.005
	PLWC ⇒ ATT	0.0139	0.0468	−0.0778	0.1056	0.0126	0.2974	0.766
	PDP ⇒ ATT	0.0404	0.0492	−0.0561	0.1368	0.0374	0.8200	0.412
	PLWLS ⇒ ATT	−0.0470	0.0494	−0.1439	0.0498	−0.0354	−0.9520	0.341
	UP ⇒ ATT	0.4482	0.0270	0.3954	0.5010	0.7192	16.6274	<0.001
	UP ⇒ BI	0.1740	0.0385	0.0985	0.2495	0.3096	4.5177	<0.001
	ATT ⇒ BI	0.4906	0.0612	0.3706	0.6106	0.5442	8.0135	<0.001
Direct	PSI ⇒ BI	0.0085	0.0630	−0.1150	0.1319	0.0075	0.1343	0.893
	PLWC ⇒ BI	−0.0158	0.0469	−0.1077	0.0761	−0.0158	−0.3363	0.737
	PDP ⇒ BI	−0.0679	0.0494	−0.1647	0.0289	−0.0699	−1.3754	0.169
	PLWLS ⇒ BI	−0.0963	0.0496	−0.1935	9.17e-4	−0.0803	−1.9415	0.052
Total	PSI ⇒ BI	0.4200	0.0827	0.2580	0.5821	0.3735	5.0801	<0.001
	PLWC ⇒ BI	0.1221	0.0657	−0.0067	0.2509	0.1226	1.8580	0.063
	PDP ⇒ BI	0.0160	0.0702	−0.1215	0.1535	0.0165	0.2280	0.820
	PLWLS ⇒ BI	−0.1188	0.0707	−0.2574	0.0199	−0.0991	−1.6790	0.093

PSI, Perceived Social Isolation; PLWC, Perceived Lack of Working Comfort; PDP, Perceived Decline in Productivity; PLWLS, Perceived Lack of Work-Life Separation; UP, Perceived Usefulness; ATT, Attitude; BI, Behavioral Intention.

#### Moderated effects

Out of the four items of perceived feasibility included in the model (Budget, Localization, Job Compatibility, Management Agreement), three proved to be significant moderators of the positive effect of Attitude on Behavioral Intention: Cost, β = 0.492, *z* = 2.529, *p* = 0.011, Job Compatibility, β = 0.176, *z* = 2.631, *p* = 0.008, and Management Agreement, β = 0.484, *z* = 3.047, *p* = 0.002. Although this relationship was not expected, Cost was a significant moderator of the positive effect of Perceived Usefulness on Behavioral Intention as well, β = 0.276, *z* = 3.332, *p* < 0.001. These results provide support for H7, H9, and H10. Conversely, Localization had no moderating impact on the positive relationship between Attitude and Behavioral Intention, β = 0.006, *z* = 0.297, *p* = 0.766, nor on any other significant paths in the model. These results invalidate H6. Despite its association with several measures included in the model (see above), Teleworking Intensity was not found to be a significant moderator of any paths tested in the model, and no interactions between the factors included as moderators were found (all *p* > 0.10).

**Figure 3:**
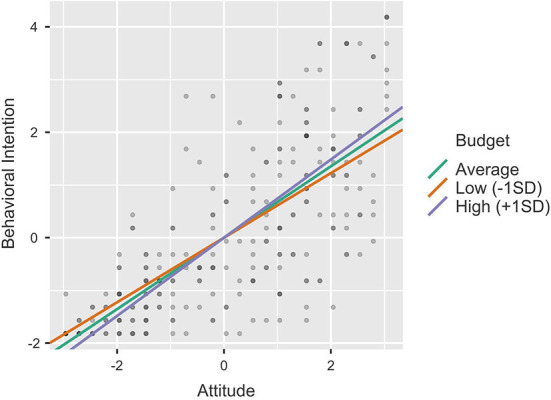
Single slope plot—Budget.

**Figure 4:**
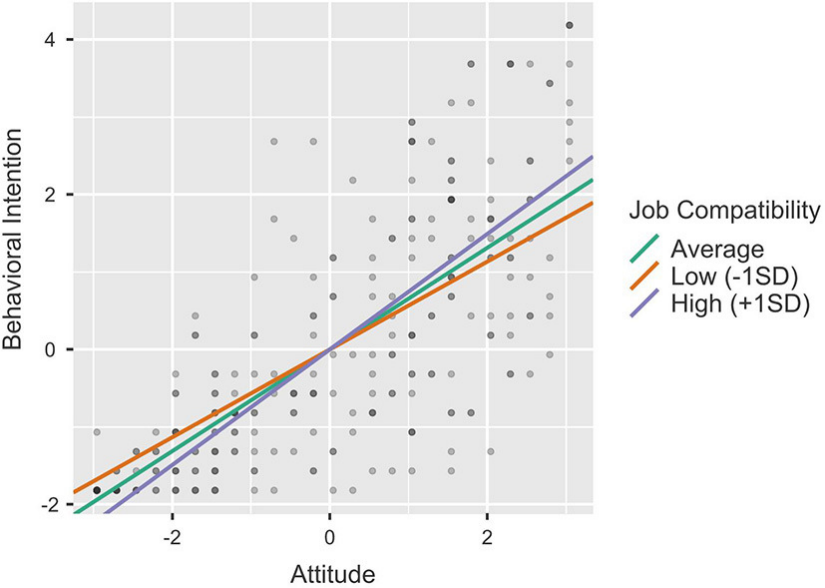
Single slope plot—Job compatibility.

**Figure 5:**
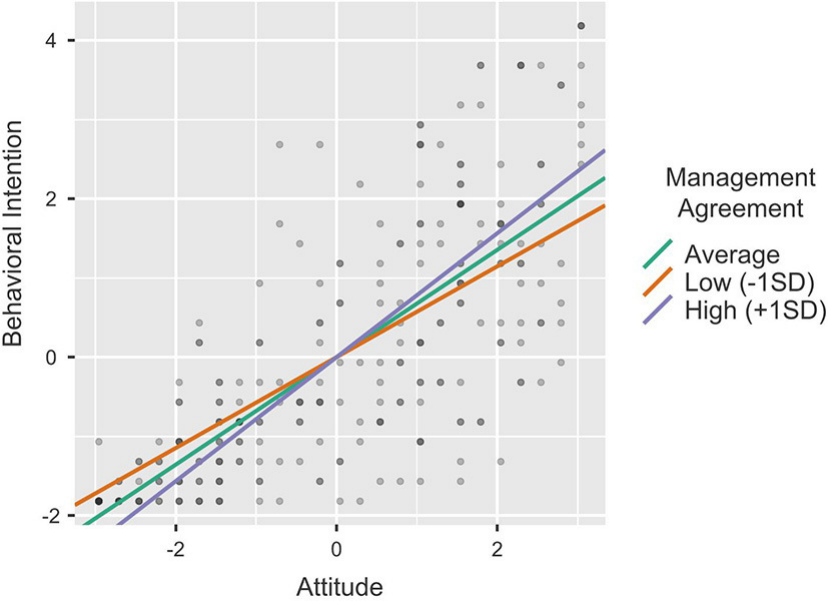
Single slope plot—Management agreement.

To clarify the moderating impact of Budget, Job Compatibility and Management Agreement on the relationship between Attitude and Behavioral Intention, we conducted single slope analyses by calculating the effect of the predictor (Attitude) on the dependent variable (Behavioral Intention) at different levels of the moderator (−1SD, average, +1SD). Table 6 provides the results of the single slope analyses and Figures 3–5 the single slope plot for each significant moderator (respectively, Budget, Job Compatibility, and Management Agreement). Overall, the pattern of results proved similar for all moderators. Although the positive effect of Attitude on Behavioral Intention remained strong and significant regardless of the moderators' levels (all *p* < 0.001), this effect was smaller when the Budget, Job Compatibility and Company Agreement scores were low (−1SD) and conversely, higher when the moderator scores were high (+1SD). These results further support H7, H9, and H10.

**Table 6:** Results of the single slope analyses.

**Moderator level**	**Estimate**	**SE**	***Z***	***p***
**Budget**				
Average	0.677	0.0359	18.9	<0.001
Low (−1SD)	0.613	0.0479	12.8	<0.001
High (+1SD)	0.742	0.0508	14.6	<0.001
**Job compatibility**				
Average	0.656	0.0360	18.2	<0.001
Low (−1SD)	0.567	0.0511	11.1	<0.001
High (+1SD)	0.745	0.0507	14.7	<0.001
**Management agreement**				
Average	0.678	0.0354	19.2	<0.001
Low (−1SD)	0.573	0.0502	11.4	<0.001
High (+1SD)	0.782	0.0489	16.0	<0.001

The analyses show the effect of the predictor (Attitude) on the dependent variable (Behavioral Intention) at different levels of the moderators.

## Discussion

This study aimed at better understanding the determinants of employees' intention to telework in a coworking space, with the assumption that employees' experience of the negative aspects of teleworking from home would impact their intention to telework in a coworking space in the future. More specifically, we expected that when employees experienced: (1) social isolation, (2) a decline in productivity, (3) a lack of working comfort, and (4) a spillover of work into their private life when working from home, they would perceive teleworking in a coworking space as more useful, have a more positive attitude toward it and thus be more inclined to telework in a coworking space in the future. However, the results of our study are not entirely in line with these assumptions.

As expected, the experience of a feeling of social isolation when working from home positively (and strongly) impacted how useful participants perceived teleworking in a coworking space to be, and indirectly how inclined they were to telework in a coworking space in the future. These results are particularly interesting in that they support the claim that perceived social isolation constitutes an important predictor of employees' intention to telework in a coworking space (Boboc et al., 2014; Gerdenitsch et al., 2016; Bianchi et al., 2018; Lashani and Zacher, 2021; Rådman et al., 2022; Wright et al., 2022). Since perceived social isolation had the strongest effect on perceived usefulness (and indirectly on behavioral intention) out of the four predictors investigated in our study, the prospect of feeling less lonely when teleworking and having more social interactions at work appears to be the main benefit perceived by employees of teleworking in a coworking space, in the same way as identified for self-employed workers (Boboc et al., 2014; Gerdenitsch et al., 2016; Lashani and Zacher, 2021; Rådman et al., 2022). As participants who teleworked more than 50% of their working hours reported a stronger feeling of social isolation than the other participants when teleworking from home, which reproduces past findings (Bailey and Kurland, 2002; Mann and Holdsworth, 2003; Gajendran and Harrison, 2007; Vayre and Pignault, 2014; Vayre, 2019), the potential benefit of teleworking in a coworking space for social integration might be all the more substantial for employees who telework intensively and may explain why these participants also reported being more inclined to telework in a coworking space in the future.

Results are much more mixed concerning the effects of the other negative aspects of teleworking from home investigated in this study. Perceived lack of working comfort impacted positively how useful teleworking in a coworking space was perceived to by the participants, and indirectly participants' intention to telework in a coworking space. However, its effect on these measures proved smaller than that of perceived social isolation, and no effect of perceived decline in productivity, nor of perceived lack of work-life separation, was observed on either perceived usefulness, attitude or intention to telework in a coworking space in the future.

The lack of effect of perceived decline of productivity might be explained by the fact that, when participants did report a decline in productivity when teleworking from home, it was for operational (e.g., problems of communication in the team) or psychological reasons (e.g., dissatisfaction with the position) that a relocation of teleworking would not solve (Vayre and Pignault, 2014; Vayre, 2019). Likewise, participants may not have perceived that teleworking in a coworking space would solve any issues related to work-life balance. The experience of a work-life spillover when teleworking may be the result of work intensification and an increase in the number of hours worked that the relocation of teleworking would not address (Bailey and Kurland, 2002; Vayre and Pignault, 2014; Vayre, 2019). A limitation of our measure of perceived lack of work-life separation (and overall, of teleworking satisfaction) is that it did not consider whether teleworking was imposed by the company. When employees encounter work-life balance issues when teleworking because being at home generates family demands (e.g., taking care of children) or distractions that interfere with work, they have the option of returning to the company premises to work, if teleworking was initially *their* decision. In situations where telework is imposed (by the company or due to compelling circumstances) and home prove to be an unsuitable place to work (Müller et al., 2022), relocating telework to a coworking space can be one of the only alternatives to work in good conditions. These assumptions warrant testing in further studies.

In addition, this study highlighted certain factors that impede employees' intention to telework in a coworking space, even if this relocation of work is perceived as beneficial: budget, job compatibility and management agreement. As assumed, these three factors proved to be significant moderators of the effect of attitude on behavioral intention, as the positive effect of attitude on behavioral intention was weaker (albeit still significant and strong) when participants felt that: (1) they did not have the budget to afford the fees to access a coworking space, (2) their job was not entirely feasible in a coworking space, and (3) their management would be reluctant for them to work in a coworking space. While the problem of job compatibility can hardly be circumvented, budget and management agreement issues might be solved by negotiation with the employers and an adjustment of company policies (provided that the company can afford to subsidize at least part of the cost of the subscription to a coworking space). This is all the more critical for employees who telework intensively. As the results of this study and past findings indicate, employees who telework intensively are more likely to experience a feeling of socio-professional isolation, detrimental to their job satisfaction, motivation and ultimately quality of life (Bailey and Kurland, 2002; Mann and Holdsworth, 2003; Gajendran and Harrison, 2007; Vayre and Pignault, 2014; Vayre, 2019). To the extent that teleworking in a coworking space can help reduce this feeling of isolation (Gerdenitsch et al., 2016; Bianchi et al., 2018; Robelski et al., 2019; Lashani and Zacher, 2021), offering coworking spaces as an alternative to the home as a place to telework seems necessary to allow this population to telework under better conditions.

### Limitations of the study and research perspectives

Although this study provides valuable insights into the determinants of employees' intention to telework in a coworking space, some limitations should be considered. Firstly, by focusing on the negative aspects of teleworking as predictors of employees' intention to telework in a coworking space, this study leaves out several factors that may also affect this intention and potentially interact with a negative experience of teleworking from home. The type of work tasks performed by employees, their satisfaction with the position they hold are some examples. Subjective norms are also known to impact behavioral intention, regardless of the behavior considered (Ajzen, 1991, 2020). Although injunctive norms (i.e., “the expectation that a given referent individual or group approves or disapproves of performing the behavior”, Ajzen, 2020, p. 315) are partly considered in the study through management agreement, the opinion of participants' colleagues regarding teleworking in a coworking space was not investigated. Prior experience with coworking spaces can also affect behavioral intention, depending on the quality of the experience (Ajzen, 2020). While frequency of use was controlled for in this study, as current users were excluded from participation, prior experience with coworking spaces was not verified. Further research is thus still needed to better understand which factors affect employees' intention to telework in a coworking space, and effectively contribute to the adoption of coworking spaces as a place to telework.

However, the question of the most appropriate model to investigate these factors remains. Technology acceptance models, such as TAM (Davis, 1989), UTAUT (Venkatesh et al., 2003), or more recently the human-technology-organization symbiosis (Brangier et al., 2010) appear inappropriate in the context of coworking spaces, in that these models were designed to investigate primarily the acceptance of the use of information systems. We chose the theory of planned behavior as a basis for this study precisely because of its lack of specificity. It is designed to predict any kind of behavior, not solely the use of a technology or an information system (Ajzen, 2020). Unfortunately, if the lack of specificity of the theory can be an advantage, it is also its main drawback. Unlike models of technology acceptance, which propose general dimensions (e.g., effort expectancy and performance expectancy) affecting attitude toward the technology studied, the theory of planned behavior does not propose dimensions affecting attitude toward a given behavior other than the very broad categories “behavioral” and “normative beliefs”. What precisely these behavioral and normative beliefs consist of must be the subject of prior pilot studies, which we have done here, but with the risk that the pilot data may not be completely representative of the actual population investigated.

Some limitations related to our study sample should also be considered. Despite our efforts to gather the largest possible sample of teleworkers, the final sample size may not have allowed us to highlight some of the interactions between the variables included in our model. Moreover, our sample proved to be very homogeneous on certain socio-demographic variables (e.g., high position in the company and high level of education). Although these characteristics of our sample are representative of the characteristics of the French teleworker population before the COVID-19 crisis (DARES, 2019), a more varied sample in terms of position in the company or level of education might have revealed some disparities in satisfaction with telework or attitudes toward teleworking in coworking spaces. Finally, some limitations related to our measures remain to be considered. If the reliability of the scales was checked before further analyses, the construct validity of our measures was not directly assessed. Yet, the addition of a general job satisfaction measure would have allowed for an assessment of the convergent validity of the telework satisfaction scale. Such measures were not included in our study, which limits the significance of the results obtained.

## Conclusion

Despite these limitations, this study is, to our knowledge, the first to have investigated the determinants of employees' intention to telework in a coworking space and, more specifically, the extent to which their experience of the negative aspects of home-based teleworking contributes to this intention. The results of the study show, in line with the literature on self-employed workers (e.g., Gerdenitsch et al., 2016), that the perception of social isolation when teleworking from home has a strong and positive impact on the perceived usefulness of teleworking in a coworking space, and indirectly on employees' intention to telework in a coworking space in the future. The perceived lack of working comfort at home also indirectly and positively affects this intention, albeit to a lesser extent. Furthermore, three factors were identified as limiting the intention to telework in a coworking space, despite a positive attitude toward this behavior: budget, management agreement and job compatibility.

This study is a first step in understanding the factors predicting employees' intention to adopt coworking spaces as a place to telework, and now needs to be complemented by further studies investigating the effect of factors other than experience of home-based teleworking as predictors of behavioral intention and final adoption. These studies are necessary to identify the needs of employees seeking alternatives to the home as a place to telework, especially employees who telework intensively and who are most likely to experience the negative aspects of teleworking at home, such as social isolation.

## Data availability statement

The raw data supporting the conclusions of this article will be made available by the authors, without undue reservation.

## Ethics statement

The studies involving human participants were reviewed and approved by Comité d'Ethique des Recherche, Université de Toulouse. The patients/participants provided their written informed consent to participate in this study.

## Author contributions

CLes, CLem, and VL conceived and designed the study. CLes analyzed the data, interpreted the results, and drafted the article. All authors contributed to the article and approved the submitted version.

## Footnotes

^1^The neutrality of the text was checked during our pilot study (Lescarret et al., 2022). We provided the description to the participants and asked them whether they considered that description negative, neutral or positive. All participants declared finding the description neutral (*N* = 17) or somewhat neutral (*N* = 3).
