# Supplementary material for: Coworking spaces vs. home: Does employees' experience of the negative aspects of working from home predict their intention to telework in a coworking space?
Source: Front Psychol. 2022 Dec 8;13:1079691. doi: 10.3389/fpsyg.2022.1079691 (PMC9773556; doi:10.3389/fpsyg.2022.1079691)
Supplement: Supplementary file 1 [file Table_1.docx]

**Appendix A: Descriptive Data**

|  | | | | | | | | | | | | | | | | | |
| --- | --- | --- | --- | --- | --- | --- | --- | --- | --- | --- | --- | --- | --- | --- | --- | --- | --- |
|  | | | | | | | | | | **Skewness** | | | | **Kurtosis** | | | |
|  | | **Mean** | | **SD** | | **Minimum** | | **Maximum** | | **Skewness** | | **SE** | | **Kurtosis** | | **SE** | |
| Perceived Social Isolation |  | 3.327 |  | 1.43 |  | 1.00 |  | 7.00 |  | 0.3149 |  | 0.149 |  | -0.668 |  | 0.297 |  |
| Perceived Lack of Comfort |  | 2.970 |  | 1.62 |  | 1.00 |  | 7.00 |  | 0.5007 |  | 0.149 |  | -0.944 |  | 0.297 |  |
| Perceived Decline in Productivity |  | 3.109 |  | 1.66 |  | 1.00 |  | 7.00 |  | 0.4770 |  | 0.149 |  | -0.803 |  | 0.297 |  |
| Perceived Lack of Work-Life Separation |  | 3.236 |  | 1.35 |  | 1.00 |  | 7.00 |  | 0.1160 |  | 0.149 |  | -0.546 |  | 0.297 |  |
| Budget |  | 2.862 |  | 1.76 |  | 1 |  | 7 |  | 0.5117 |  | 0.149 |  | -0.962 |  | 0.297 |  |
| Localization |  | 4.317 |  | 1.85 |  | 1 |  | 7 |  | -0.2019 |  | 0.149 |  | -0.948 |  | 0.297 |  |
| Management Agreement |  | 4.000 |  | 1.97 |  | 1 |  | 7 |  | 0.0416 |  | 0.149 |  | -1.139 |  | 0.297 |  |
| Job Compatibility |  | 5.220 |  | 1.63 |  | 1 |  | 7 |  | -0.7790 |  | 0.149 |  | -0.169 |  | 0.297 |  |
| Perceived Usefulness |  | -0.828 |  | 2.87 |  | -4 |  | 4 |  | 0.3595 |  | 0.149 |  | -1.357 |  | 0.297 |  |
| Attitude |  | 3.957 |  | 1.79 |  | 1.00 |  | 7.00 |  | -0.0692 |  | 0.149 |  | -1.226 |  | 0.297 |  |
| Behavioral Intention |  | 2.817 |  | 1.61 |  | 1.00 |  | 7.00 |  | 0.7491 |  | 0.149 |  | -0.431 |  | 0.297 |  |
|  | | | | | | | | | | | | | | | | | |
