# Supplementary material for: Coworking spaces vs. home: Does employees' experience of the negative aspects of working from home predict their intention to telework in a coworking space?
Source: Front Psychol. 2022 Dec 8;13:1079691. doi: 10.3389/fpsyg.2022.1079691 (PMC9773556; doi:10.3389/fpsyg.2022.1079691)
Supplement: Supplementary file 2 [file Table_2.docx]

|  | | | | | | | | | | | | | | | | | | | | | | | | | | |
| --- | --- | --- | --- | --- | --- | --- | --- | --- | --- | --- | --- | --- | --- | --- | --- | --- | --- | --- | --- | --- | --- | --- | --- | --- | --- | --- |
|  |  |  |  |  |  |  |  |  |  |  |  |  |  |  |  |  |  |  |  |  |  |  |  |  |  |  |
|  | |  | | **PLSI** | | **PLP** | | **PLC** | | **PLWLS** | | **Budget** | | **Localization** | | **Job Compatibility** | | **Management Agreement** | | **PU** | | **Attitude** | | **BI** | |  |
| PLSI |  | Pearson's r |  | — |  |  |  |  |  |  |  |  |  |  |  |  |  |  |  |  |  |  |  |  |  |  |
| PLP |  | Pearson's r |  | 0.601*** |  | — |  |  |  |  |  |  |  |  |  |  |  |  |  |  |  |  |  |  |  |  |
| PLC |  | Pearson's r |  | 0.471*** |  | 0.485*** |  | — |  |  |  |  |  |  |  |  |  |  |  |  |  |  |  |  |  |  |
| PLWLS |  | Pearson's r |  | 0.325*** |  | 0.181** |  | 0.263*** |  | — |  |  |  |  |  |  |  |  |  |  |  |  |  |  |  |  |
| Budget |  | Pearson's r |  | 0.030 |  | 0.074 |  | -0.061 |  | 0.006 |  | — |  |  |  |  |  |  |  |  |  |  |  |  |  |  |
| Localization |  | Pearson's r |  | 0.073 |  | 0.125* |  | 0.094 |  | 0.004 |  | -0.015 |  | — |  |  |  |  |  |  |  |  |  |  |  |  |
| Job Compatibility |  | Pearson's r |  | 0.051 |  | 0.076 |  | 0.054 |  | 0.091 |  | 0.050 |  | 0.329*** |  | — |  |  |  |  |  |  |  |  |  |  |
| Management Agreement |  | Pearson's r |  | -0.040 |  | 0.060 |  | -0.062 |  | -0.072 |  | 0.206*** |  | 0.201*** |  | 0.251*** |  | — |  |  |  |  |  |  |  |  |
| PU |  | Pearson's r |  | 0.560*** |  | 0.434*** |  | 0.429*** |  | 0.202*** |  | 0.054 |  | 0.204*** |  | 0.223*** |  | 0.019 |  | — |  |  |  |  |  |  |
| Attitude |  | Pearson's r |  | 0.558*** |  | 0.433*** |  | 0.395*** |  | 0.165** |  | 0.026 |  | 0.315*** |  | 0.187** |  | -0.031 |  | 0.811*** |  | — |  |  |  |  |
| BI |  | Pearson's r |  | 0.409*** |  | 0.282*** |  | 0.281*** |  | 0.058 |  | 0.134* |  | 0.432*** |  | 0.240*** |  | 0.136* |  | 0.702*** |  | 0.750*** |  | — |  |  |
| Note : *: p <.05; **: p <.01; ***: p <.001 | | | | | | | | | | | | | | | | | | | | | | | | | | |

**Appendix B: Correlations between measures**
